# Antitoxin

**Published:** 1895-06

**Authors:** 


Antitoxin.—Doubtless it is yet too early to declare that the efficiency of the antitoxin treatment has been demonstrated, but it must be said that the prospect of such a demonstration is apparent. Whenever any inquiry of this sort is before the profession, and especially when it is before the public, as in this instance, there are never wanting those who set their faces with the fixity of fanaticism against the measure, can see no good in anything so novel and radical, and seize upon every misadventure in its employment to confirm and justify their opposition. —*New York Medical Journal.*

